# UNC-Emory Infant Atlases for Macaque Brain Image Analysis: Postnatal Brain Development through 12 Months

**DOI:** 10.3389/fnins.2016.00617

**Published:** 2017-01-10

**Authors:** Yundi Shi, Francois Budin, Eva Yapuncich, Ashley Rumple, Jeffrey T. Young, Christa Payne, Xiaodong Zhang, Xiaoping Hu, Jodi Godfrey, Brittany Howell, Mar M. Sanchez, Martin A. Styner

**Affiliations:** ^1^Department of Psychiatry, University of North CarolinaChapel Hill, NC, USA; ^2^Kitware Inc.Carrboro, NC, USA; ^3^Department of Computer Science, University of North CarolinaChapel Hill, NC, USA; ^4^Division of Autism and Related Developmental Disabilities, Department of Pediatrics, Marcus Autism Center, Children's Healthcare of Atlanta, Emory School of MedicineAtlanta, GA, USA; ^5^Yerkes National Primate Research Center, Emory UniversityAtlanta, GA, USA; ^6^Department of Bioengineering, University of California, RiversideRiverside, CA, USA; ^7^Department of Child Psychology, Institute of Child Development, University of MinnesotaMinneapolis, MN, USA; ^8^Department of Psychiatry and Behavioral Sciences, Emory UniversityAtlanta, GA, USA

**Keywords:** neuroimaging, non-human primate, macaque, computational atlases, white matter pathways, magnetic resonance imaging, diffusion tensor imaging, automatic segmentation

## Abstract

Computational anatomical atlases have shown to be of immense value in neuroimaging as they provide age appropriate reference spaces alongside ancillary anatomical information for automated analysis such as subcortical structural definitions, cortical parcellations or white fiber tract regions. Standard workflows in neuroimaging necessitate such atlases to be appropriately selected for the subject population of interest. This is especially of importance in early postnatal brain development, where rapid changes in brain shape and appearance render neuroimaging workflows sensitive to the appropriate atlas choice. We present here a set of novel computation atlases for structural MRI and Diffusion Tensor Imaging as crucial resource for the analysis of MRI data from non-human primate rhesus monkey (*Macaca mulatta*) data in early postnatal brain development. Forty socially-housed infant macaques were scanned longitudinally at ages 2 weeks, 3, 6, and 12 months in order to create cross-sectional structural and DTI atlases via unbiased atlas building at each of these ages. Probabilistic spatial prior definitions for the major tissue classes were trained on each atlas with expert manual segmentations. In this article we present the development and use of these atlases with publicly available tools, as well as the atlases themselves, which are publicly disseminated to the scientific community.

## Introduction

Non-human primate models are widely used for comparative studies with human neuropathology (Lebherz et al., 2005; Lubach and Coe, 2006; Short et al., 2010; Shi et al., 2013). Advantages include the biological similarity, such as the gestation of a single offspring, a prolonged *in utero* development, and the maturational stage of the neonatal brain at birth. Among non-human primate models, the rhesus macaque (*Macaca mulatta*) has been the most widely studied monkey due to its phylogenetic closeness to humans (Rilling and Insel, 1999; Passingham, 2009) and the potential to examine more complex functions and behavior associated with encephalization (Price and Coe, 2000). The use of monkeys in a socially complex but captive setting like that at the Yerkes National Primate Research Center (YNPRC) focus of this paper also facilitates species-typical experiences while ensuring genetically and environmentally controlled studies (Harlow et al., 1971). Additionally, rhesus macaques show hemispheric asymmetry and sex differences in their brains during adolescence similar to humans (Shi et al., 2013).

While there are detailed neuroanatomical, and neurochemical descriptions of early postnatal maturation in some regions of the monkey brain, such as the amygdala, hippocampus and prefrontal and visual cortices (Goldman-Rakic, 1987; Hoftman and Lewis, 2011; Chareyron et al., 2012), less information exist on global brain maturation, with the exception of early studies of cortical circuitry and synaptogenesis (Rakic et al., 1986; Goldman-Rakic, 1987).

MRI studies have significantly expanded our knowledge of brain development during childhood (Sowell et al., 2002; Shaw et al., 2008; Knickmeyer et al., 2010; Gilmore et al., 2012; Shi et al., 2013). Image-based reference atlases (templates) are crucial to provide information for automatic processing of MRI individual data. These atlases are used for aligning new images for the purpose of normalization into a common coordinate space (Mazziotta et al., 2001). In addition, regions of interest can be outlined in the template images and propagated from these to the observed data (Fonov et al., 2011; Kuklisova-Murgasova et al., 2011) for segmentation or region analysis.

Currently a number of structural and diffusion MRI-based macaque brain atlases are available, mainly from adults (Styner et al., 2007; McLaren et al., 2009; Rohlfing et al., 2012; Calabrese et al., 2015) and elderly (Adluru et al., 2012) rhesus macaque populations. There is though a notable lack of publically available macaque atlases during the early postnatal brain development. This paper fills that void providing with MRI atlases for structural and diffusion MRI analysis at postnatal ages that expand the infant and early juvenile periods (postnatal ages 2 weeks, 3, 6, and 12 months).

## Materials and methods

In this section, we describe the methods and data used to construct the early brain development atlases and their associated maps. For that purpose, we employed a number of image analysis software tools, most of them available as open source (see Section Referenced Resources for a full list) for intensity bias correction, image alignment, group-wise registration, deformable image registration, tissue segmentations, reformatting, diffusion image processing, and general-purpose image operations.

### Subjects

This study was conducted at the Yerkes National Primate Research Center (YNPRC) Field Station, Emory University (Lawrenceville, GA). A total of 40 infant rhesus monkeys (*M. mulatta*) were used for the generation of the atlases as part of a larger study where animals were scanned longitudinally during infancy and the early juvenile period (from 2 weeks through 18 months of age) to examine the role of maternal care on brain and biobehavioral development (see Hoftman and Lewis, 2011; McCormack et al., 2015; Howell et al., 2017). Subject age and gender distribution at each age is shown in Table 1. The infants lived with their mothers for the entire duration of the study and families in large social groups consisting of 75–150 adult females, their sub-adult and juvenile offspring and 2–3 males. The groups were housed in outdoor compounds (~100 × 100 ft) with adjacent climate-controlled indoor housing area. Standard high fiber, low fat monkey chow (Purina Mills Int., Lab Diets, St. Louis, MO) and seasonal fruits and vegetables were provided twice daily, in parallel to enrichment items. Water was available *ad libitum*. All studies were performed in accordance with the Animal Welfare Act and the U.S. Department of Health and Human Services “Guide for the Care and Use of Laboratory Animals,” and approved by the Emory University Institutional Animal Care and Use Committee (IACUC).

**Table 1:** **Subject table**.

	**2 weeks**	**3 months**	**6 months**	**12 months**	**18 months**
Age (days)	15 ± 4	84 ± 4	171 ± 6	368 ± 7	535 ± 6
Number of scans	34	36	37	40	35
Sex (m/f)	16/18	19/17	20/17	21/19	18/17

In this work, we present atlases that represent typically developing, socially housed rhesus monkeys with a broad range of maternal care experiences and also spanning all social hierarchy strata (high, medium, and low ranking families). Atlases were generated at ages 2 weeks, 3, 6, and 12 months. In our experiments, 18 month old structural datasets were well-represented by the 12 month atlas and thus no additional 18 month structural atlas was generated.

### Imaging

**Figure 1:**
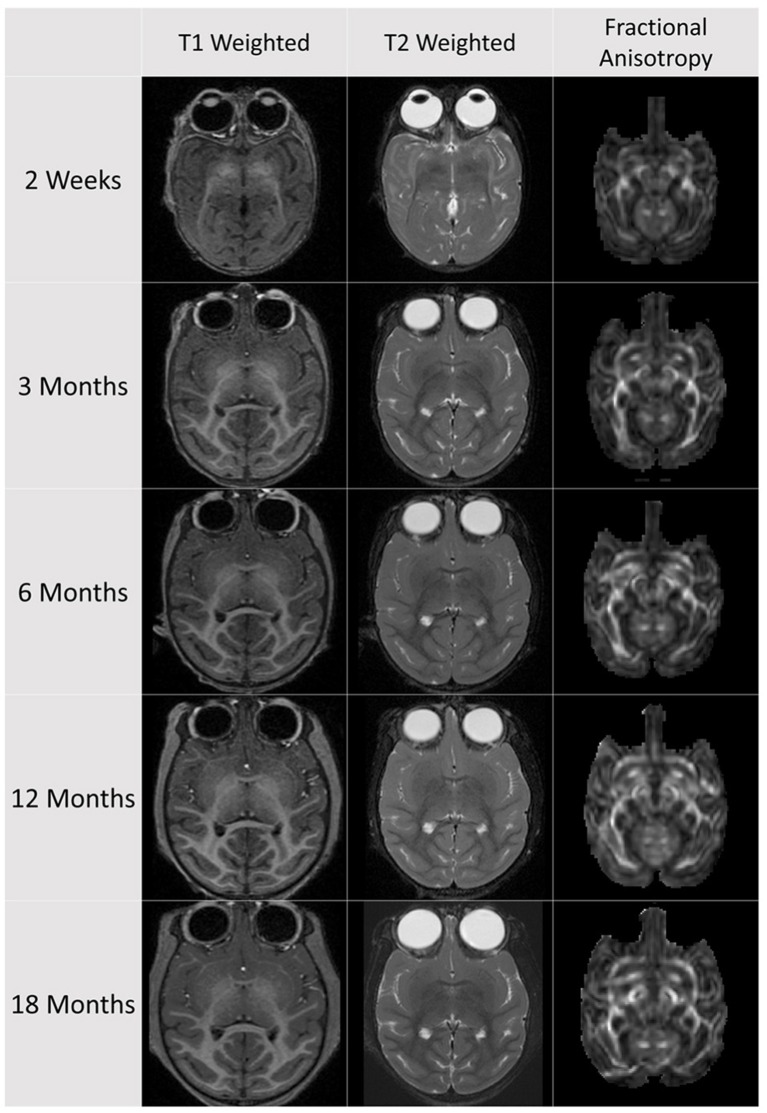
**Example subject dataset with scans from 2 weeks to 18 months of age**. At each age a T1 weighted and T2 weighted scan, as well as a diffusion weighted/tensor scan was acquired. The diffusion data has been skull stripped and up-interpolated to isotropic 0.65 mm resolution.

T1 weighted (T1w), T2 weighted (T2w), and diffusion weighted images (DWI) were acquired longitudinally at ages 2 weeks, 3, 6, 12, and 18 months (see Figure 1 for an example subject scanset). Thirty-one subjects had a full set of five longitudinal scans; eight subjects had four successful scan sessions, and one subject had three successful scan sessions. Images were acquired on a 3T Siemens Trio scanner (Malvern, PA) at the Yerkes National Primate Research Center's (YNPRC) Imaging Center using an eight-channel array, transmit and receive knee volume coil. The subjects were scanned supine under isoflurane anesthesia (0.8–1% isoflurane, inhalation). A custom-made head holder with ear bars and a mouth piece was used to secure and prevent movement of the head in order to avoid motion artifacts. A vitamin E capsule was placed to the right temple to identify the right brain hemisphere. Animals were intubated, administered dextrose/NaCl (I.V.) for hydration, placed over an MRI-compatible heating pad to maintain temperature and physiological measures monitored during the scans. After each subject was scanned and had completely recovered from anesthesia, it was returned to its mother, and mother-infant dyad returned to their social group.

T1w data were acquired with a magnetization prepared rapid gradient echo (MPRAGE) sequence with the following parameters: TI/TR/TE = 950/3000/3.3 ms; four averages; flip angle 8, voxel size: 0.6 mm^3^ isotropic resolution, FOV 115.2 × 115.2 mm, matrix 192 × 192). T2w images were collected in the same direction as the T1 (TR/TE = 7900/125 ms, voxel size = 0.5 × 0.5 × 1.0 mm^3^, 10 averages, FOV 96 × 96 mm, matrix 192 × 192) to help with anatomical identification of white matter (WM), gray matter (GM) and cerebrospinal fluid (CSF) borders, and delineation of ROIs (Knickmeyer et al., 2010). DWI data were collected with a single-shot dual spin-echo EPI sequence with GRAPPA (*R* = 3), voxel size: 1.3 × 1.3 × 1.3 mm^3^ with zero gap, 60 directions, TR/TE = 5000/86 ms, 40 slices, FOV: 83 × 83 mm, matrix 64 × 64, b:0, 1000 s/mm^2^, and 12 averages, acquisition time: 75 min.

### Structural MRI atlas building

#### Pre-processing

Before the atlas generation process, we pre-processed all datasets in the following way, using the AutoSeg version 3.3.2, segmentation package (Wang et al., 2014): First, all MRI datasets were converted from DICOM into the volumetric NRRD format (Fedorov et al., 2012), followed by intensity inhomogeneity correction using the N4 (Tustison et al., 2010) tool and rigid body registration to a prior external atlas space, the UNC-Wisconsin juvenile macaque atlas, created from 18 cases of rhesus macaques aged 16–34 months (Styner et al., 2007)^1^, via AutoSeg (v3.3.2). Rigid registration to the prior atlas is computed for the T1w images, whereas each T2w image is registered to is corresponding atlas-aligned T1w image. Due to this initial registration, the resulting atlases are approximately rigidly aligned with the UNC-Wisconsin juvenile macaque atlas space. Both individual T1w and T2w were subsequently resampled to the juvenile atlas resolution at isotropic 0.273 mm. For the generation of the initial brain mask (for the purpose of skull stripping), we employed the UNC-Wisconsin juvenile macaque atlas via ABC (Prastawa et al., 2005). All these initial, automatically generated brain masks were manually corrected by human expert raters via InsightSNAP (Yushkevich et al., 2006).

#### Cross-sectional atlas building

**Figure 2:**
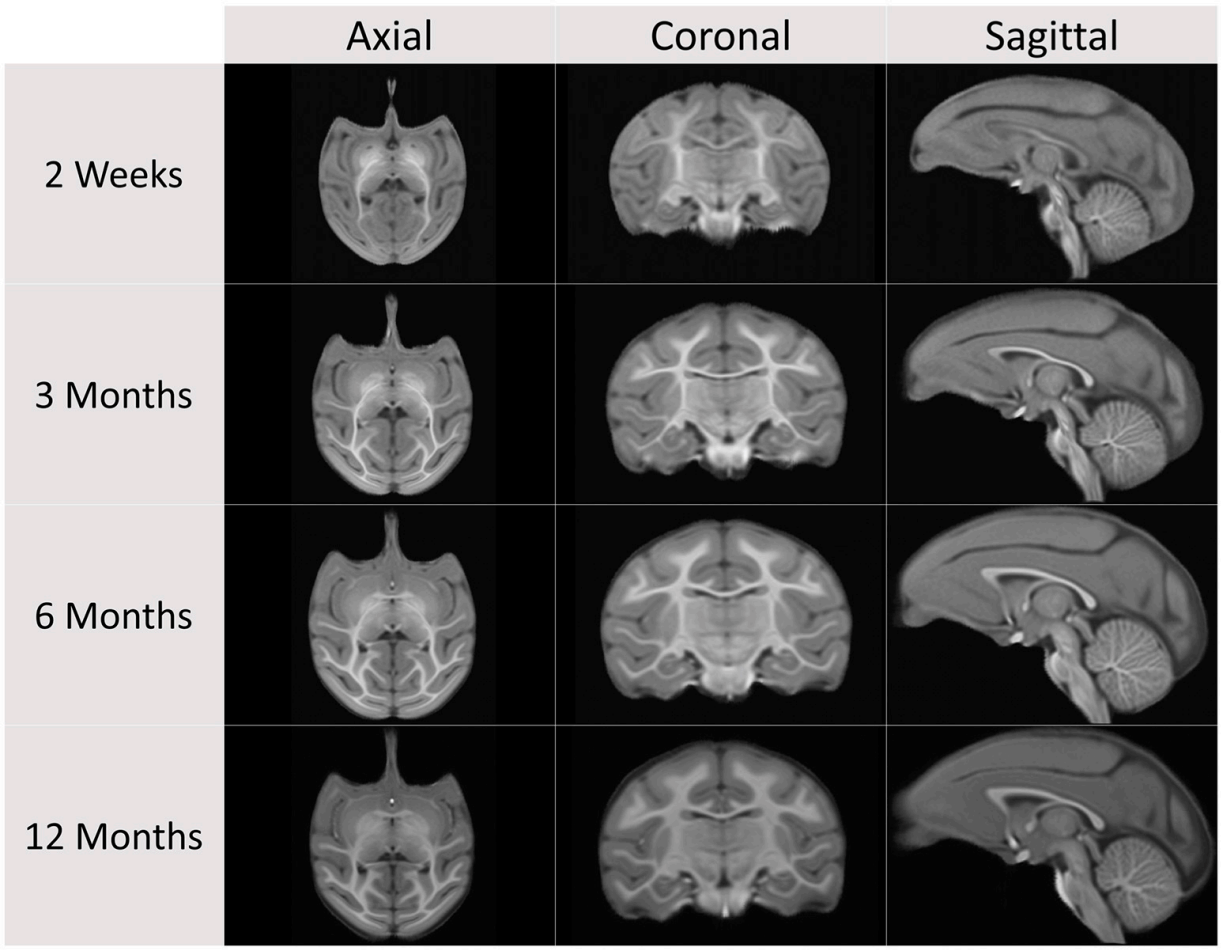
**Cross-sectional average T1-weighted atlas images at the four atlas building ages**.

**Figure 3:**
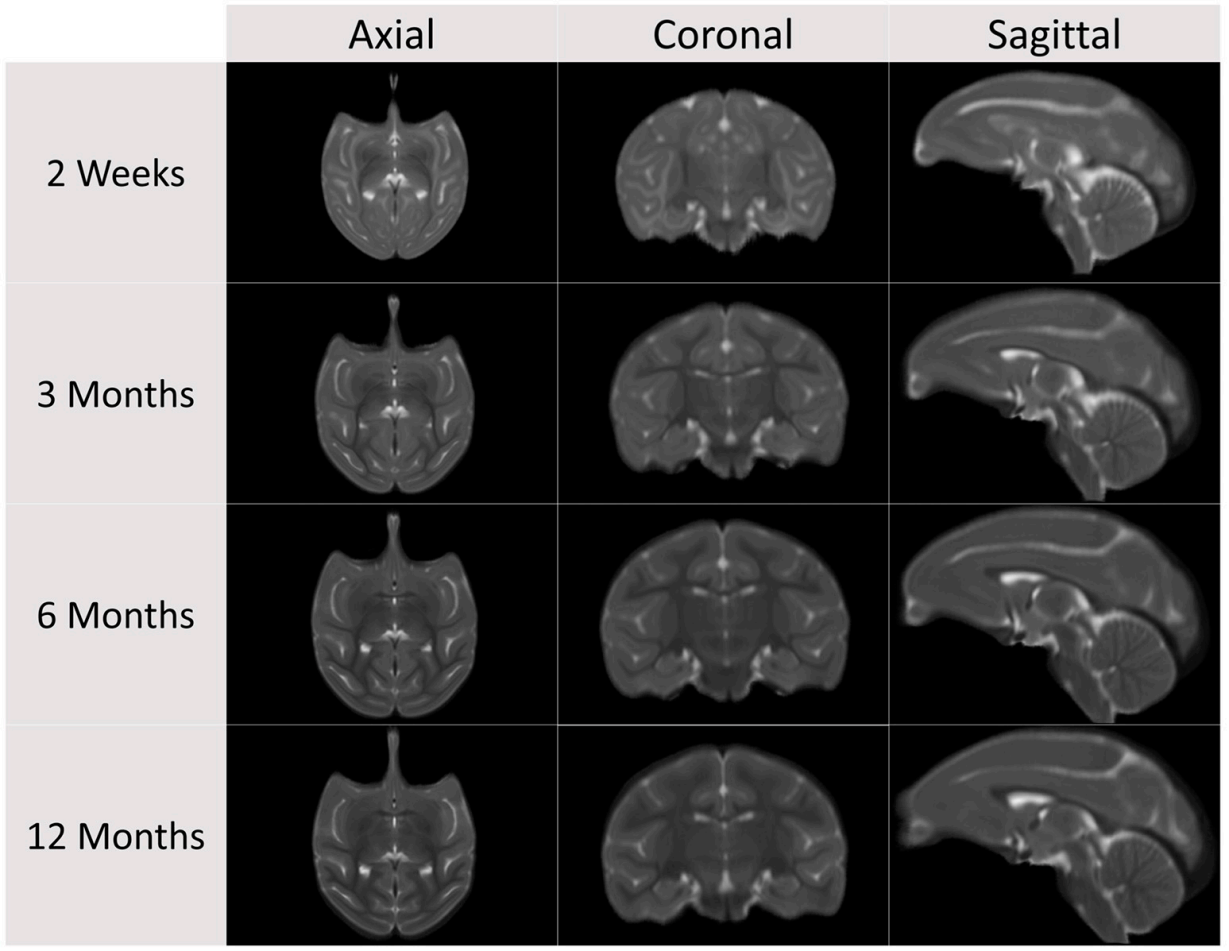
**Cross-sectional average T2-weighted atlas images at the four atlas building ages**.

**Figure 4:**
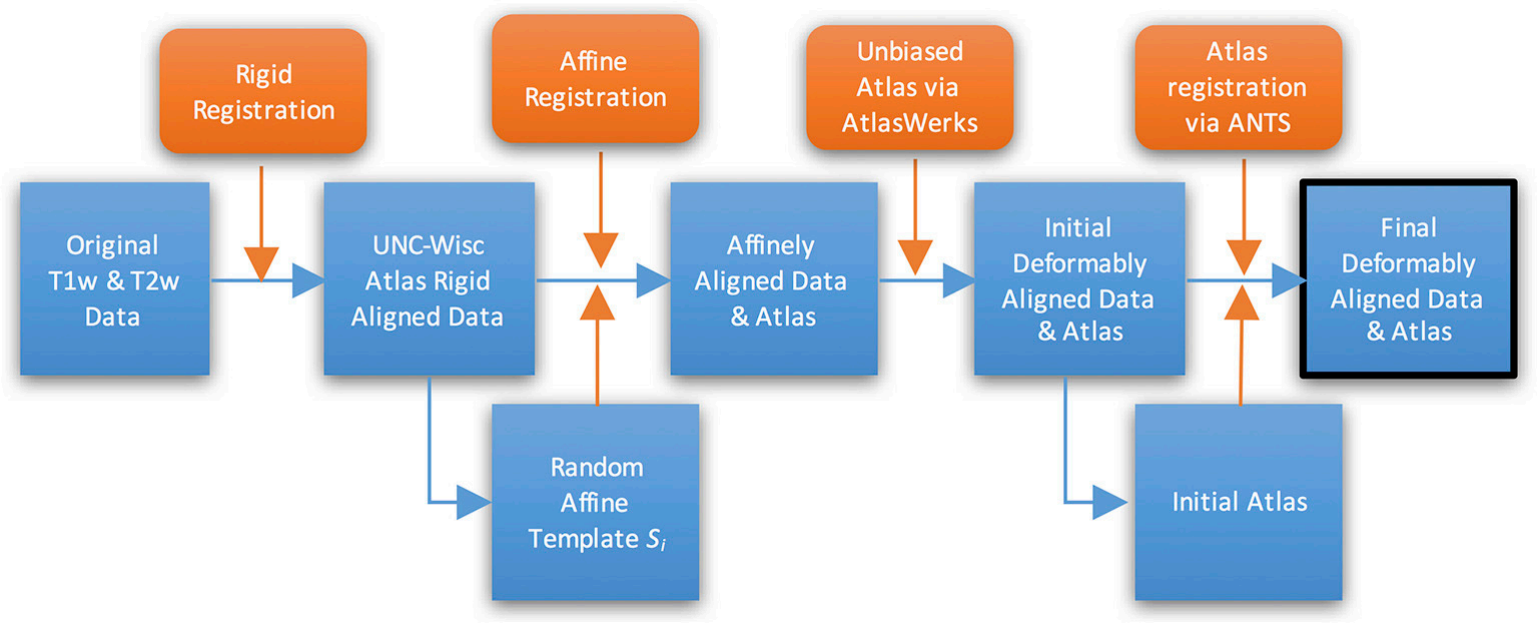
**Schematic view of atlas building workflow**.

**Figure 5:**
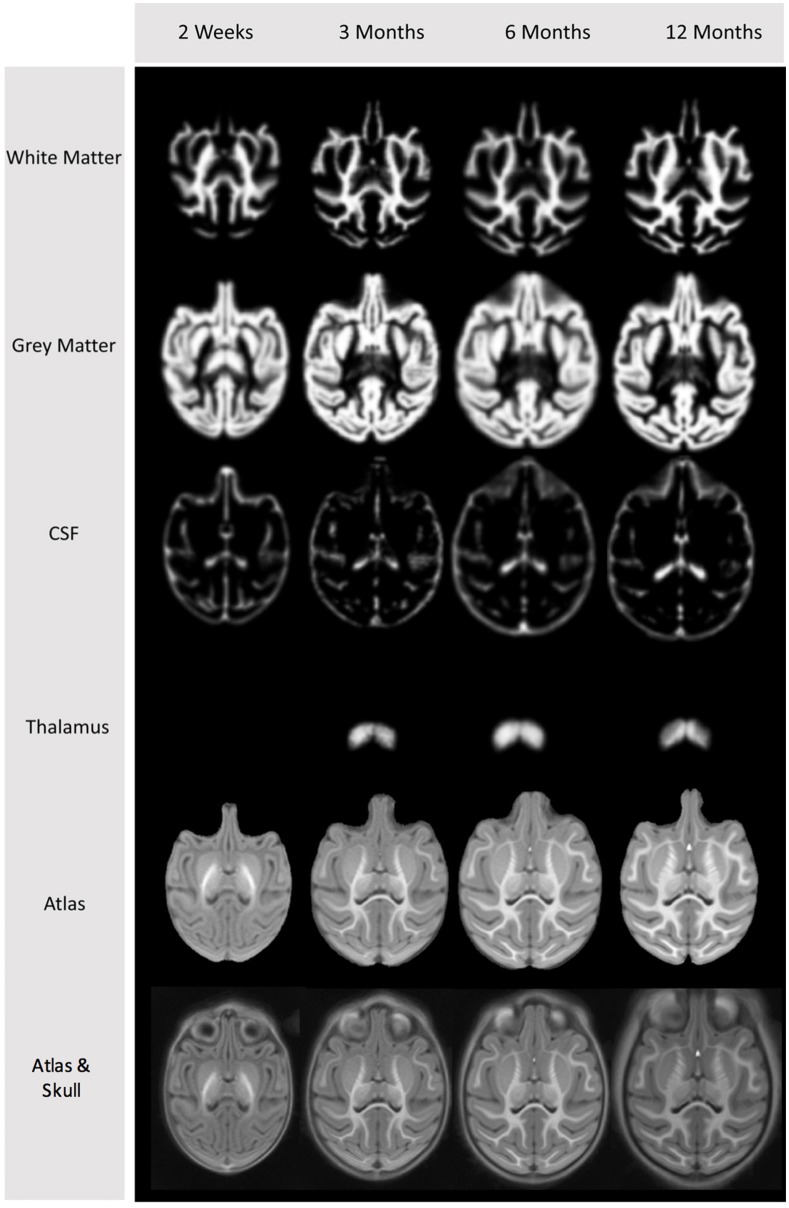
**Tissue segmentation priors in atlas space at the four different ages**.

Cross-sectional structural population average atlas images were generated at ages 2 weeks, 3, 6, and 12 months (see Figures 2, 3), independently, employing the following steps starting with the pre-processed, brain masked images (see Figure 4 for a schematic of the atlas building workflow and Figure 5 for the resulting structural atlases):

1. A subject *S*_*t*_ was randomly chosen as a template for intensity normalization (step 2) and initial affine alignment (step 3).
2. All T1w and T2w images were intensity normalized via histogram-quantile based intensity calibration to the T1w and T2w images of *S*_*t*_.
3. Initial affine alignment of all T1w images to *S*_*t*_'s T1w image was performed next via the general registration module in 3D Slicer (Fedorov et al., 2012). All aligned T1w images were averaged to generate the structural affine atlas *A*_*affine*_. A final affine alignment was then computed for all T1w images to *A*_*affine*_. The resulting affine transforms were applied to the corresponding T2w images.
4. An initial unbiased T1w atlas image was constructed from the affinely aligned T1w images (Joshi et al., 2004) using unbiased diffeomorphic fluid-model based deformable registration with AtlasWerks yielding deformation fields that warp individual T1w images into the atlas space *A*_*def*_. These deformation fields were then applied to the T2w images and an unbiased T2w atlas image in the same atlas space *A*_*def*_ was computed.
5. Building upon this initial deformable atlas images, we constructed a more refined final atlas *A*_*final*_ by registering all individual images to *A*_*def*_ using a multi-channel (equally weighted), symmetric deformable registration via ANTS (Avants et al., 2011; metric: cross-correlation at kernel radius of 1 mm), followed by averaging the registered images. A final deformable alignment for all images was performed by applying the same ANTS based registration to *A*_*final*_.
6. The final deformation fields that warp subjects to the atlas space *A*_*final*_ were applied to non-skull-stripped data to compute non-skull-stripped atlas images.

All registration steps in this atlas building were assessed for appropriate mapping of the individual MR images into the atlas space via MriWatcher (See Supplemental Figure 1).

#### Tissue segmentation priors on the atlas

We computed prior probability maps for a standard tissue segmentation of white matter (WM), gray matter (GM), and CSF. These prior maps can be used with most tissue segmentation tools as part of AutoSeg (Wang et al., 2014), ANTS (Avants et al., 2011), and FSL (Jenkinson et al., 2012). The prior maps were determined in two phases, first iteratively for the 12 month old data, which were then propagated to the younger ages. These prior probability maps were based on manually corrected tissue segmentations from four subjects only and not the whole atlas population. Consequently, while the atlas grayscale images are of unbiased nature, the prior probability maps are biased to the (random) selection of these four subjects.

##### 12 month atlas and tissue class priors

1. We first propagated manually corrected tissue type label segmentation from an existing adolescent-age structural atlas (Knickmeyer et al., 2010) to the 12 month cross-sectional atlas space via symmetric deformable registration via ANTS (Avants et al., 2011). This was performed using the T1w images only. The propagated tissue segmentation was then manually corrected by experienced raters. In addition, new tissue types for thalamus, globus pallidus (GP), and large vessels were manually outlined in atlas space to improve the segmentation results, as vessels appears of similar intensity to WM on the T1w scans. We then performed dilation of one voxel followed by a Gaussian smoothing (sigma.1, iteration 5) separately for each tissue class. Finally, all the segmentation probability maps were normalized and a background-rejection class prior was computed. We will refer to this atlas and priors as atlas *A*_*12-1*_.
2. Automatic segmentation was performed via ABC, a deformable registration based atlas moderated Expectation Maximization (EM) classification approach provided within AutoSeg (version 3.3.2), with atlas *A*_*12-1*_ on all the subjects. We then manually corrected all segmentation tissue labels on four randomly chosen subjects.
3. The *A*_*12-1*_ tissue probability maps were then updated with the four manually segmented cases by mapping the segmentations back into atlas space, where they were averaged, Gaussian smoothed (sigma.1), and re-normalized to generate priors we will refer to as *A*_*12-2*_.
4. The *A*_*12-2*_ tissue probability maps were iteratively optimized. In each optimization loop, we first computed the automatic tissue segmentation via ABC (as in step 2) of the four cases, then determined difference/error maps of the automatic segmentation vs. the manual segmentation, ranging from −1 to 1 for each tissue type. Next, this error map was propagated back to the atlas space and the probability map for each tissue class were corrected by subtracting the corresponding average error map multiplied by 0.25. The corrected probability maps were then renormalized. This procedure was iteratively performed until convergence of the error maps, yielding the final tissue class probability maps *A*_*12*_.

##### 3 and 6 month atlas and tissue class priors

We selected the same four subjects as for generation of *A*_*12*_ and applied automatic tissue segmentation via ABC to the scans at 3 and 6 months of age with *A*_*12*_ as the prior atlas. Tissue segmentation was performed The segmentation results of these four subjects were then manually corrected and the same optimization steps (3) and (4) as for *A*_*12*_ were applied to yield age specific priors atlases *A*_*3*_ and *A*_*6*_.

##### 2 week atlas and tissue class priors

Similar to above, we selected the same four subjects and applied automatic tissue segmentation to the scans at 2 weeks of age with *A*_3_ as the prior atlas. The segmentation results of these four subjects were then manually corrected and the same optimization steps (3) and (4) as for *A*_*12*_ were applied to yield age specific priors atlases *A*_*2w*_.

##### Subcortical region definition

Subcortical region label maps were propagated from the existing UNC-Wisconsin adolescent atlas (Styner et al., 2007) to the 12 month atlas image by deformable registration via ANTS (Avants et al., 2011). The subcortical label maps were then manually edited and propagated to 6, 3 month and 2 week atlas via deformable co-registration.

##### Parcellations

Lobar regional parcellation maps were propagated in the same fashion as the subcortical regions from the existing UNC-Wisconsin adolescent atlas first to the 12 month atlas and then to other ages.

##### Histological regions

We also co-registered the high resolution post-mortem atlas with regional definitions according to the Paxinos histological atlas (Calabrese et al., 2015) using a multi-modality (equally weighted), symmetric deformable registration via ANTS (Avants et al., 2011). Those regional definitions were propagated to the 12 month atlas.

### DWI and DTI quality control

#### DWI quality control—DTIPrep

Diffusion-weighted images were up-interpolated to an isotropic 0.65 mm resolution using windowed sinc interpolation. Removal of artifact rich diffusion weighted images, as well as correction of motion and eddy current artifacts were performed with the in-house tool DTIPrep (Oguz et al., 2014; Liu et al., 2015) via affine registration of diffusion-weighted images to the baseline B0 image. Diffusion tensors were computed using weighted least squares fitting (Goodlett et al., 2007). The tensor Eigenvalues were calculated to obtain diffusion property maps, including fractional anisotropy (FA), mean diffusivity (MD), axial diffusivity (AD), and radial diffusivity (RD). Skull stripping was performed after propagation of the manual structural mask via deformable registration of the structural T2 weighted image to the corresponding B0 images.

### DTI atlas building

The diffusion atlas generated by the atlas building described below is defined approximately in the general space as the structural atlases, the juvenile UNC-Wisconsin atlas space. It is noteworthy though that the diffusion atlases and the structural atlases are not voxel-wise corresponding.

#### Cross-sectional DTI atlas

A preliminary FA atlas was created first at 12 months. The same basic algorithm used for the structural atlas computation was used after applying histogram normalization to all FA maps yielding diffeomorphic field maps that warp each individual subject to the atlas.

**Figure 6:**
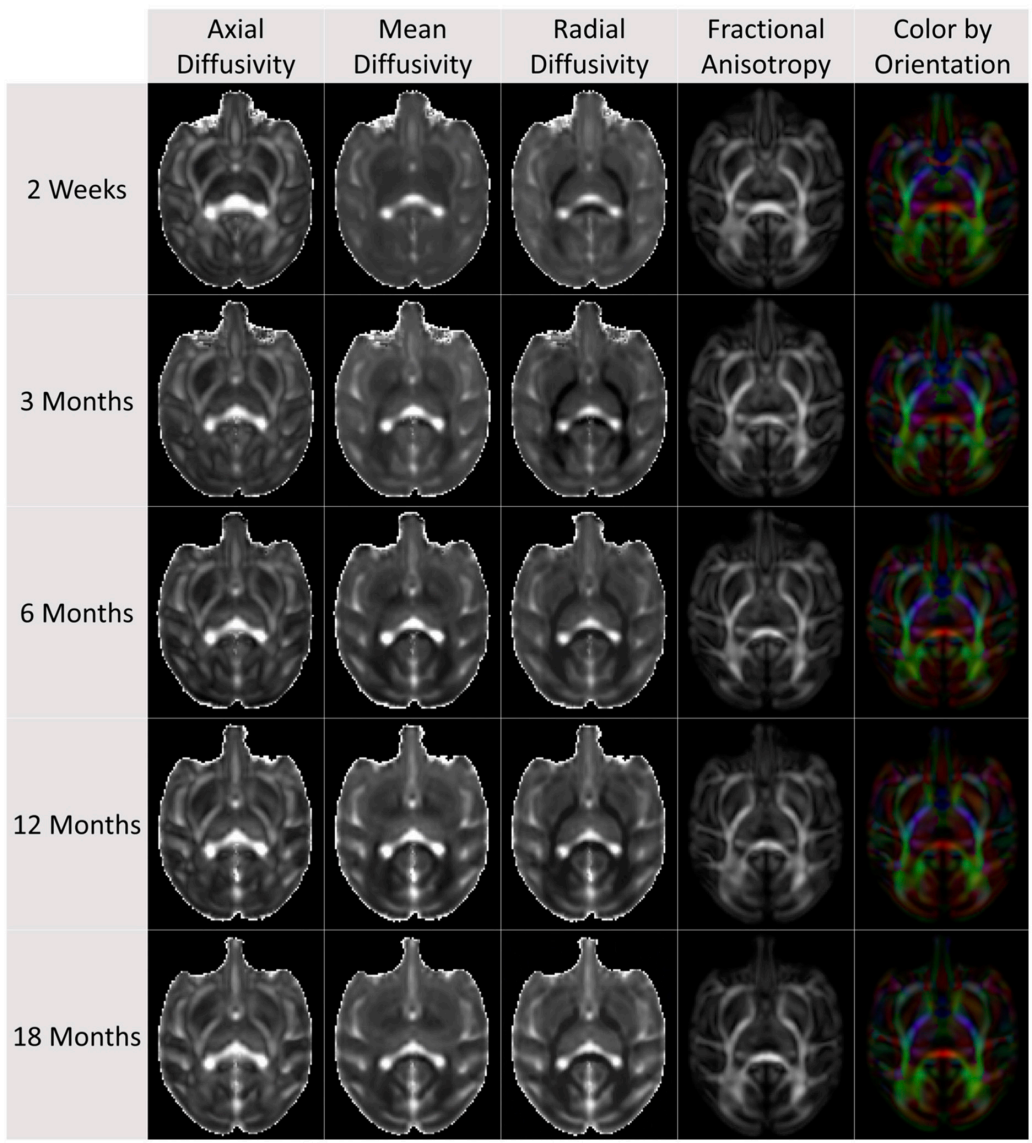
**Cross-sectional average DTI atlas images at the five atlas building ages on the same axial slice for AD, MD, RD, FA, and orientation-colored FA**.

This FA map was then used as the affine template for creating cross-sectional atlases at 2 weeks, 3, 6, 12, and 18 months of age. In-house tool DTIAtlasBuilder was used for atlas creation in this step. In detail, at each age, all the FA maps were first affine registered to a prior FA template. For 12 months, that template was a randomly selected case, rigidly realigned to the juvenile UNC-Wisconsin T2w atlas. All other ages employed the 12 month DTI atlas as a prior template for the affine registration. Then similar to the structural atlas building process, an unbiased diffeomorphic atlas was created using AtlasWerks. Subsequently, all the FA images were registered to this diffeomorphic atlas with ANTS. The final ANTS atlas was generated by averaging the deformed individuals. The result field maps were then applied to each corresponding tensor image and a finite-strain algorithm was adopted to reorient the tensors (Alexander et al., 2001). The final DTI atlas was computed as the average over all the warped tensor images (Goodlett et al., 2009). The DTI atlases at all ages are visualized in Figure 6.

#### Longitudinal DTI atlas

A longitudinal DTI atlas was also created with all the subjects at all ages (from 2 weeks through 18 months). First, a subject-specific DTI atlas was created with images at all the time points for each subject, respectively via DTIAtlasBuilder. The initial affine template was chosen the same for all subject, specifically the same template image as for the cross-sectional 12 month atlas building, which had been rigidly aligned to the juvenile UNC-Wisconsin atlas space. The resulting subject-specific DTI atlases were then used as input for DTIAtlasBuilder to generate a longitudinal DTI atlas and displacement fields that map the subject-specific atlas to the final longitudinal space. Displacement fields from the two steps were concatenated to generate the final displacement field maps that warp the individual DTI maps to the final longitudinal atlas apace. The final displacement field maps were then applied to the original individual DTI to avoid repeated interpolation from both steps. The deformed DTI were then averaged to generate the final DTI atlas to avoid interpolation from both steps.

#### Fiber tracking

We performed fiber tracking of major rhesus monkey tracts in the DTI atlas space with 3D Slicer (Verde et al., 2014). Whole brain tractography (see Figure 9) was performed to visually confirm the presence of all major fiber tracts in the atlas DTI images. ROI seeding voxels were manually determined and standard streamline tractography was used to obtain the major fiber tracts, including the uncinate fasciculus, cingulum bundle, inferior longitudinal fasciculus, middle longitudinal fasciculus, different subdivisions of the internal capsule and genu and splenium of the corpus callosum. Tracts were identified using anatomical landmarks for the rhesus monkey brain as previously defined (Schmahmann et al., 2007; Schmahmann and Pandya, 2009).

## Applications

### Structural analysis

The structural atlases developed here (Figure 5) with their tissue type priors can be used to generate automatic tissue segmentation with any major software platform, though these have been specifically trained for use with AutoSeg.

**Figure 7:**
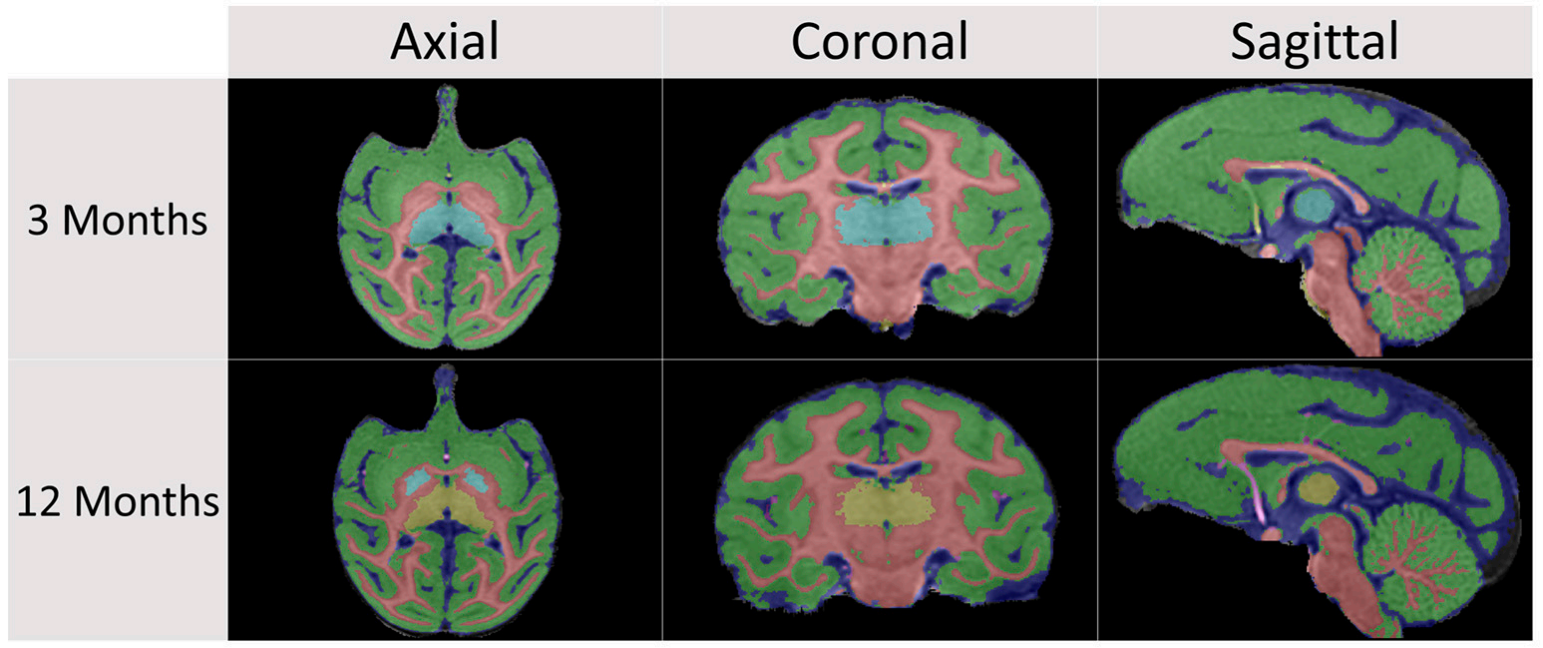
**AutoSeg tissue segmentation results in a representative subject at 3 and 12 months of age**.

**Figure 8:**
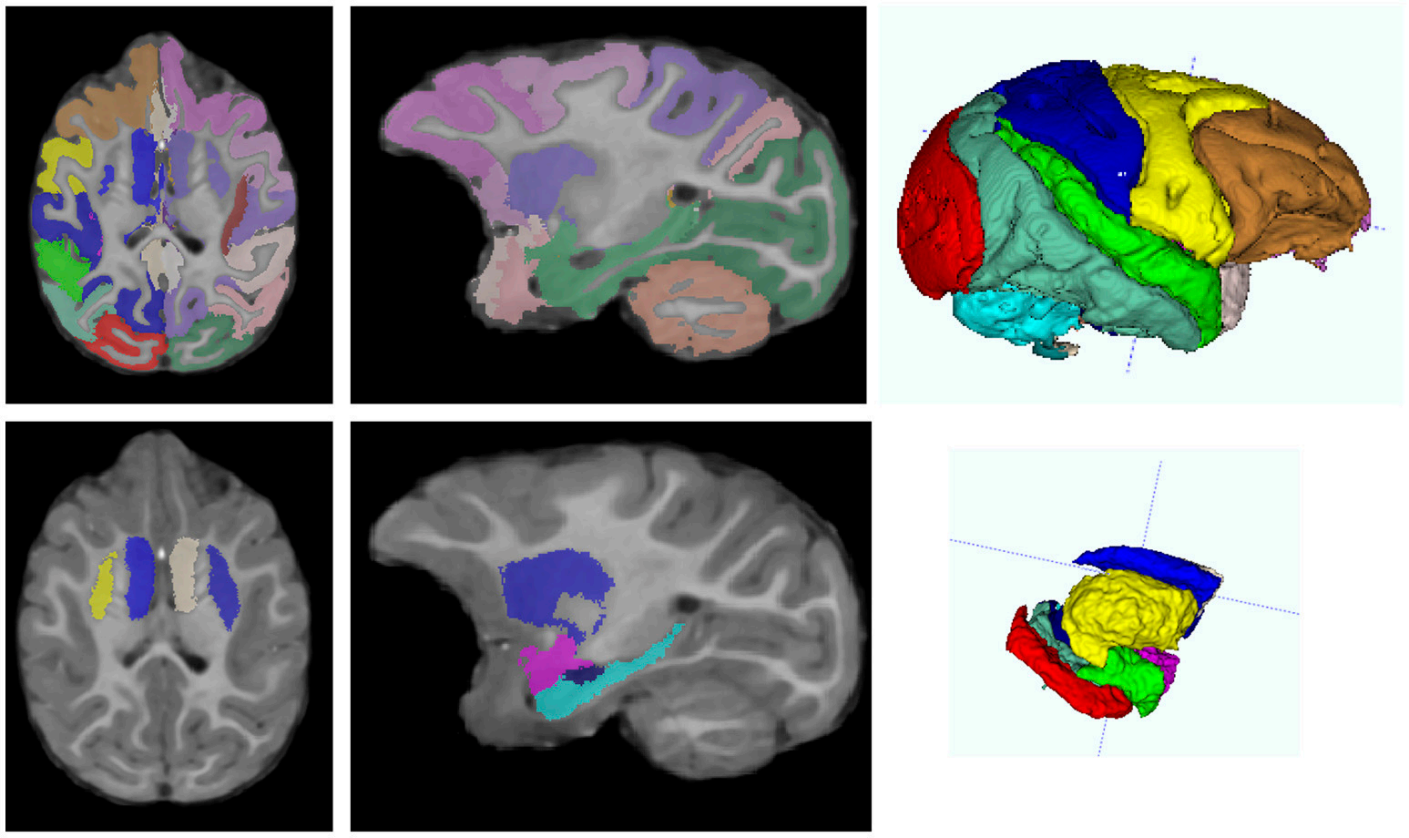
**AutoSeg GM parcellation (top row) and subcortical structures (bottom row) in a representative subject at 12 months, with axial (left)** and sagittal slice **(middle)**, and 3D rendering **(right)** views.

Figures 7, 8 show an example subject at 3 and 12 months of age segmented via AutoSeg using the structural atlas at corresponding ages. Tissue segmentation, lobar parcellation, and subcortical segmentation results can thus be automatically generated for volumetric analysis in developmental macaque studies.

**Figure 9:**
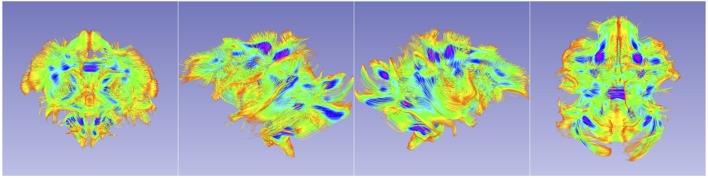
**Whole brain tractography in the 12 months cross-sectional atlas space**.

### DTI analysis

The generated longitudinal DTI atlases can be used as a reference space for analysis of developmental macaque data, as well as the provided fiber tracts in atlas space can be used for fiber based analysis, such as in Shi et al. (2013) and Verde et al. (2014). In our own studies, we employ these atlases both as reference spaces as well as fiber definitions.

## Conclusion

In this manuscript, we present a novel resource for the analysis of macaque MRI datasets in early postnatal brain development. Both structural and diffusion based atlases were generated from at ages 2 weeks to 12 months (and additionally at age 18 months for the DTI atlases). These atlas images alongside the supporting information of tissue class priors and fiber tracts are being disseminated publically as a general resource for the field.

For the use of our atlases in diffusion analyses, we recommend the use of the age-appropriate cross-sectional atlas for cross-sectional studies and the longitudinal atlas for longitudinal studies in this early postnatal phase.

All generated atlases described in this paper are available at the following Neuro Imaging Toolkit and Resource Clearinghouse (NITRC) webpage: https://www.nitrc.org/projects/macaque_atlas/ (Note: Atlases will be publically released upon publication of this manuscript).

## Referenced resources

**3D Slicer**: http://www.slicer.org

**DTIPrep**: http://www.nitrc.org/projects/dtiprep/

**AutoSeg**: http://www.nitrc.org/projects/autoseg/

**itk-SNAP**: http://www.itksnap.org/pmwiki/pmwiki.php

**DTIAtlasBuilder**: http://www.nitrc.org/projects/dtiatlasbuilder/

**MriWatcher**: http://www.nitrc.org/projects/mriwatcher/

**AtlasWerks**: http://www.sci.utah.edu/software/13/370-atlaswerks.html

**DTI-Reg**: http://www.nitrc.org/projects/dtireg/

**BRAINS**: http://www.nitrc.org/projects/brains/

**ANTS**: http://www.picsl.upenn.edu/ANTS/

**FiberViewerLight**: http://www.nitrc.org/projects/fvlight/

**DTIAtlasFiberAnalyzer**: http://www.nitrc.org/projects/dti_tract_stat

**FADTTS**: http://www.nitrc.org/projects/fadtts/

**Matlab**: http://www.mathworks.com/products/matlab/

## Author contributions

Study Design: MMS. Methods: MAS, YS. Project oversight: MAS, MMS. MR protocol design and acquisition: XZ, XH. Animal handling: CP, JG, BH. Initial data processing, quality control: YS, BH. Manual edits of brain masks and tissue segmentations: EY, AR, CP, JG. Atlas generation: YS, FB. Writing: YS, JY, MAS, MMS. Figure generation: YS, JY, MAS.

### Conflict of interest statement

The authors declare that the research was conducted in the absence of any commercial or financial relationships that could be construed as a potential conflict of interest.

## Footnotes

^1^https://www.nitrc.org/projects/primate_atlas

## References

[B1] Adluru N. Zhang H. Fox A. S. Shelton S. E. Ennis C. M. Bartosic A. M.. (2012). A diffusion tensor brain template for Rhesus Macaques. NeuroImage 59, 306–318. 10.1016/j.neuroimage.2011.07.029 21803162 PMC3195880

[B2] Alexander D. C. Pierpaoli C. Basser P. J. Gee J. C. (2001). Spatial transformations of diffusion tensor magnetic resonance images. IEEE Trans. Med. Imaging 20, 1131–1139. 10.1109/42.963816 11700739

[B3] Avants B. B. Tustison N. J. Song G. Cook P. A. Klein A. Gee J. C. (2011). A reproducible evaluation of ANTs similarity metric performance in brain image registration. NeuroImage 54, 2033–2044. 10.1016/j.neuroimage.2010.09.025 20851191 PMC3065962

[B4] Calabrese E. Badea A. Coe C. L. Lubach G. R. Shi Y. Styner M.. (2015). A diffusion tensor MRI atlas of the postmortem rhesus macaque brain. NeuroImage 117, 408–416. 10.1016/j.neuroimage.2015.05.072 26037056 PMC4512905

[B5] Chareyron L. J. Lavenex P. B. Lavenex P. (2012). Postnatal development of the amygdala: a stereological study in rats. J. Comp. Neurol. 520, 3745–3763. 10.1002/cne.23132 22523001

[B6] Fedorov A. Beichel R. Kalpathy-Cramer J. Finet J. Fillion-Robin J. C. Pujol S.. (2012). 3D Slicer as an image computing platform for the Quantitative Imaging Network. Magn. Reson. Imaging 30, 1323–1341. 10.1016/j.mri.2012.05.001 22770690 PMC3466397

[B7] Fonov V. Evans A. C. Botteron K. Almli C. R. McKinstry R. C. Collins D. L.. (2011). Unbiased average age-appropriate atlases for pediatric studies. NeuroImage 54, 313–327. 10.1016/j.neuroimage.2010.07.033 20656036 PMC2962759

[B8] Gilmore J. H. Shi F. Woolson S. L. Knickmeyer R. C. Short S. J. Lin W.. (2012). Longitudinal development of cortical and subcortical gray matter from birth to 2 years. Cereb. Cortex 22, 2478–2485. 10.1093/cercor/bhr327 22109543 PMC3464410

[B9] Goldman-Rakic P. S. (1987). Development of cortical circuitry and cognitive function. Child Dev. 58, 601–622. 10.2307/1130201 3608641

[B10] Goodlett C. B. Fletcher P. T. Gilmore J. H. Gerig G. (2009). Group analysis of DTI fiber tract statistics with application to neurodevelopment. NeuroImage 45, S133–S142. 10.1016/j.neuroimage.2008.10.060 19059345 PMC2727755

[B11] Goodlett C. B. Fletcher P. T. Lin W. Gerig G. (2007). Quantification of measurement error in DTI: theoretical predictions and validation. Med. Image Comput. Comput. Assist. Interv. 10, 10–17. 10.1007/978-3-540-75757-3_2 18051038

[B12] Harlow H. F. Harlow M. K. Suomi S. J. (1971). From thought to therapy: lessons from a primate laboratory. Am. Sci. 59, 538–549. 5004085

[B13] Hoftman G. D. Lewis D. A. (2011). Postnatal developmental trajectories of neural circuits in the primate prefrontal cortex: identifying sensitive periods for vulnerability to schizophrenia. Schizophr. Bull. 37, 493–503. 10.1093/schbul/sbr029 21505116 PMC3080694

[B14] Howell B. R. McMurray M. S. Guzman D. B. Nair G. Shi Y. McCormack K. M.. (2017). Maternal buffering beyond glucocorticoids: impact of early life stress on corticolimbic circuits that control infant responses to novelty. Soc. Neurosci. 12, 50–64. 10.1080/17470919.2016.1200481 27295326 PMC5585074

[B15] Jenkinson M. Beckmann C. F. Behrens T. E. Woolrich M. W. Smith S. M. (2012). FSL. NeuroImage 62, 782–790. 10.1016/j.neuroimage.2011.09.015 21979382

[B16] Joshi S. Davis B. Jomier M. Gerig G. (2004). Unbiased diffeomorphic atlas construction for computational anatomy. NeuroImage 23, S151–S160. 10.1016/j.neuroimage.2004.07.068 15501084

[B17] Knickmeyer R. C. Styner M. Short S. J. Lubach G. R. Kang C. Hamer R.. (2010). Maturational trajectories of cortical brain development through the pubertal transition: unique species and sex differences in the monkey revealed through structural magnetic resonance imaging. Cereb. Cortex 20, 1053–1063. 10.1093/cercor/bhp166 19703936 PMC2852502

[B18] Kuklisova-Murgasova M. Aljabar P. Srinivasan L. Counsell S. J. Doria V. Serag A.. (2011). A dynamic 4D probabilistic atlas of the developing brain. NeuroImage 54, 2750–2763. 10.1016/j.neuroimage.2010.10.019 20969966

[B19] Lebherz C. Maguire A. M. Auricchio A. Tang W. Aleman T. S. Wei Z.. (2005). Nonhuman primate models for diabetic ocular neovascularization using AAV2-mediated overexpression of vascular endothelial growth factor. Diabetes 54, 1141–1149. 10.2337/diabetes.54.4.1141 15793254

[B20] Liu B. Zhu T. Zhong J. (2015). Comparison of quality control software tools for diffusion tensor imaging. Magn. Reson. Imaging 33, 276–285. 10.1016/j.mri.2014.10.011 25460331

[B21] Lubach G. R. Coe C. L. (2006). Preconception maternal iron status is a risk factor for iron deficiency in infant rhesus monkeys (*Macaca mulatta*). J. Nutr. 136, 2345–2349. 16920852 10.1093/jn/136.9.2345

[B22] Mazziotta J. Toga A. W. Evans A. Fox P. Lancaster J. Zilles K.. (2001). A probabilistic atlas reference system for the human brain: International Consortium for Brain Mapping (ICBM). Philos. Trans. R Soc. Lond. B Biol Sci. 356, 1293–1322. 10.1098/rstb.2001.0915 11545704 PMC1088516

[B23] McCormack K. Howell B. R. Guzman D. Villongco C. Pears K. Kim H.. (2015). The development of an instrument to measure global dimensions of maternal care in rhesus macaques (*Macaca mulatta*). Am. J. Primatol. 77, 20–33. 10.1002/ajp.22307 25066041 PMC4276463

[B24] McLaren D. G. Kosmatka K. J. Oakes T. R. Kroenke C. D. Kohama S. G. Matochik J. A.. (2009). A population-average MRI-based atlas collection of the rhesus macaque. NeuroImage 45, 52–59. 10.1016/j.neuroimage.2008.10.058 19059346 PMC2659879

[B25] Oguz I. Farzinfar M. Matsui J. Budin F. Liu Z. Gerig G.. (2014). DTIPrep: quality control of diffusion-weighted images. Front. Neuroinform. 8:4. 10.3389/fninf.2014.00004 24523693 PMC3906573

[B26] Passingham R. (2009). How good is the macaque monkey model of the human brain? Curr. Opin. Neurobiol. 19, 6–11. 10.1016/j.conb.2009.01.002 19261463 PMC2706975

[B27] Prastawa M. Gilmore J. H. Lin W. Gerig G. (2005). Automatic segmentation of MR images of the developing newborn brain. Med. Image Anal. 9, 457–466. 10.1016/j.media.2005.05.007 16019252

[B28] Price K. C. Coe C. L. (2000). Maternal constraint on fetal growth patterns in the rhesus monkey (*Macaca mulatta*): the intergenerational link between mothers and daughters. Hum. Reprod. 15, 452–457. 10.1093/humrep/15.2.452 10655322

[B29] Rakic P. Bourgeois J. P. Eckenhoff M. F. Zecevic N. Goldman-Rakic P. S. (1986). Concurrent overproduction of synapses in diverse regions of the primate cerebral cortex. Science 232, 232–235. 10.1126/science.3952506 3952506

[B30] Rilling J. K. Insel T. R. (1999). The primate neocortex in comparative perspective using magnetic resonance imaging. J. Hum. Evol. 37, 191–223. 10.1006/jhev.1999.0313 10444351

[B31] Rohlfing T. Kroenke C. D. Sullivan E. V. Dubach M. F. Bowden D. M. Grant K. A.. (2012). The INIA19 template and neuromaps atlas for primate brain image parcellation and spatial normalization. Front. Neuroinform. 6:27. 10.3389/fninf.2012.00027 23230398 PMC3515865

[B32] Schmahmann J. D. Pandya D. (2009). Fiber Pathways of the Brain. Oxford: OUP.

[B33] Schmahmann J. D. Pandya D. N. Wang R. Dai G. D'Arceuil H. E. de Crespigny A. J.. (2007). Association fibre pathways of the brain: parallel observations from diffusion spectrum imaging and autoradiography. Brain 130, 630–653. 10.1093/brain/awl359 17293361

[B34] Shaw P. Kabani N. J. Lerch J. P. Eckstrand K. Lenroot R. Gogtay N.. (2008). Neurodevelopmental trajectories of the human cerebral cortex. J. Neurosci. 28, 3586–3594. 10.1523/JNEUROSCI.5309-07.2008 18385317 PMC6671079

[B35] Shi Y. Short S. J. Knickmeyer R. C. Wang J. Coe C. L. Niethammer M.. (2013). Diffusion tensor imaging-based characterization of brain neurodevelopment in primates. Cereb. Cortex 23, 36–48. 10.1093/cercor/bhr372 22275483 PMC3513950

[B36] Short S. J. Lubach G. R. Karasin A. I. Olsen C. W. Styner M. Knickmeyer R. C.. (2010). Maternal influenza infection during pregnancy impacts postnatal brain development in the rhesus monkey. Biol. Psychiatry 67, 965–973. 10.1016/j.biopsych.2009.11.026 20079486 PMC3235476

[B37] Styner M. Knickmeyer R. Joshi S. Coe C. Short S. J. Gilmore J. (2007). Automatic brain segmentation in rhesus monkeys, in SPIE Medical Imaging 2007: Image Processing (San Diego, CA), 65122L-8 10.1117/12.710027

[B38] Sowell E. R. Trauner D. A. Gamst A. Jernigan T. L. (2002). Development of cortical and subcortical brain structures in childhood and adolescence: a structural MRI study. Dev. Med. Child Neurol. 44, 4–16. 10.1017/S0012162201001591 11811649

[B39] Tustison N. J. Avants B. B. Cook P. A. Zheng Y. Egan A. Yushkevich P. A.. (2010). N4ITK: improved N3 bias correction. IEEE Trans. Med. Imaging 29, 1310–1320. 10.1109/TMI.2010.2046908 20378467 PMC3071855

[B40] Verde A. R. Budin F. Berger J. B. Gupta A. Farzinfar M. Kaiser A.. (2014). UNC-Utah NA-MIC framework for DTI fiber tract analysis. Front. Neuroinform. 7:51. 10.3389/fninf.2013.00051 24409141 PMC3885811

[B41] Wang J. Vachet C. Rumple A. Gouttard S. Ouziel C. Perrot E.. (2014). Multi-atlas segmentation of subcortical brain structures via the AutoSeg software pipeline. Front. Neuroinform. 8:7. 10.3389/fninf.2014.00007 24567717 PMC3915103

[B42] Yushkevich P. A. Piven J. Hazlett H. C. Smith R. G. Ho S. Gee J. C.. (2006). User-guided 3D active contour segmentation of anatomical structures: significantly improved efficiency and reliability. NeuroImage 31, 1116–1128. 10.1016/j.neuroimage.2006.01.015 16545965

