# Supplementary material for: UNC-Emory Infant Atlases for Macaque Brain Image Analysis: Postnatal Brain Development through 12 Months
Source: Front Neurosci. 2017 Jan 10;10:617. doi: 10.3389/fnins.2016.00617 (PMC5222830; doi:10.3389/fnins.2016.00617)
Supplement: Supplementary file 1 [file Image1.pdf]

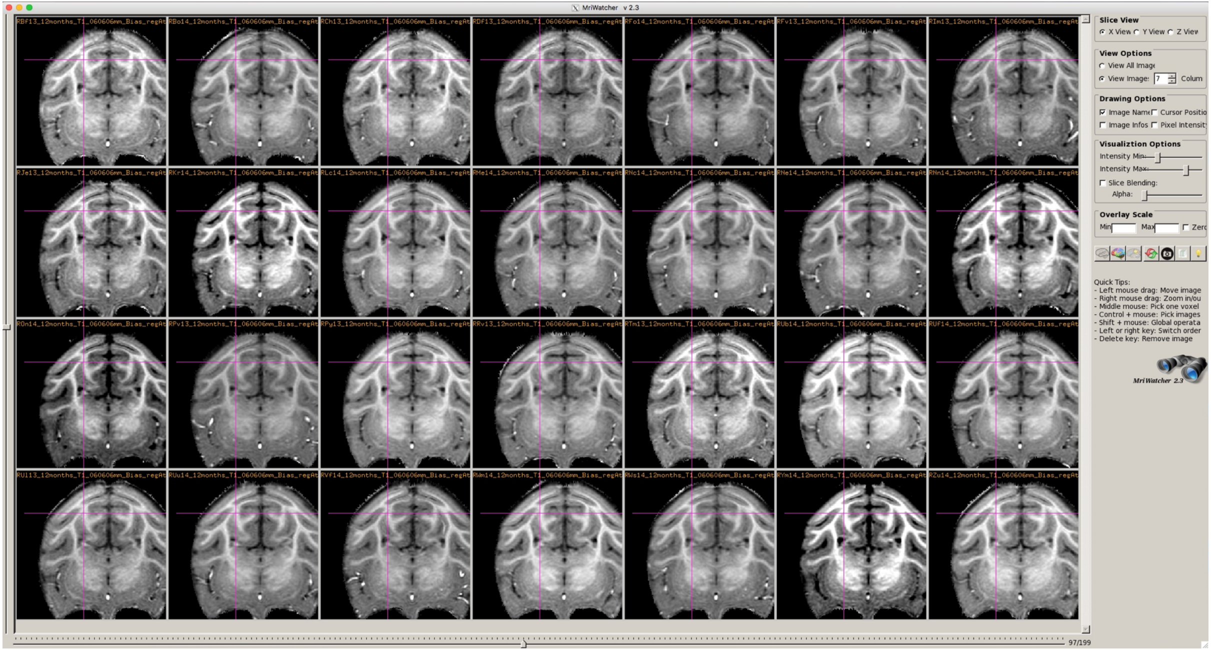

Supplemental Figure 1: Illustrative visualization of the visual QC after atlas building via MriWatcher. The tool allows the coordinated visualization of multiple images including a corresponding cross hair to enable intuitive checking of the atlas registration result.
